# Vitamin C partially attenuates male reproductive deficits in hyperglycemic rats

**DOI:** 10.1186/1477-7827-9-100

**Published:** 2011-07-27

**Authors:** Glaura SA Fernandes, Carla DB Fernandez, Kleber E Campos, Débora C Damasceno, Janete A Anselmo-Franci, Wilma DG Kempinas

**Affiliations:** 1 Graduate Program in Cell and Structural Biology, Institute of Biology, University of Campinas - UNICAMP, Campinas, SP, Brazil; 2 Department of Gynecology and Obstetrics, Botucatu Medical School, UNESP - Univ Estadual Paulista, Botucatu, SP, Brazil; 3 Department of Morphology, Stomatology and Physiology, Dental School of Ribeirão Preto, University of São Paulo - USP, Ribeirão Preto, SP, Brazil; 4 Department of Morphology, Institute of Biosciences, UNESP - Univ Estadual Paulista, Botucatu, SP, Brazil

## Abstract

**Background:**

Hyperglycemia can impair the male reproductive system in experimental animals and in men during reproductive age. Studies have shown that vitamin C has some good effects on male reproductive system, and therefore vitamin C treatment could attenuate the dysfunctions in this system caused by hyperglycemia. Thus, the objective of this work was to evaluate whether vitamin C treatment could attenuate reproductive dysfunctions in hyperglycemic male rats.

**Methods:**

Adult male rats were divided into 3 groups: a normoglycemic (n = 10) and two hyperglycemic (that received a single dose of streptozotocin - 40 mg/kg BW). The two last groups (n = 10 per group) were divided into: hyperglycemic control (Hy) and hyperglycemic + 150 mg of vitamin C (HyC), by gavage during 30 consecutive days. The normoglycemic and hyperglycemic control groups received the vehicle (water). The first day after the treatment, the rats were anesthetized and killed to evaluate oxidative stress biomarkers (TBARS, SOD, GSHt and GSH-Px) in the erythrocytes, body and reproductive organ weights, sperm parameters, plasma hormone levels (FSH, LH and testosterone), testicular and epididymal histo-morphometry and histopathology.

**Results:**

Compared with the normoglycemic animals, hyperglycemic control rats showed reduced weight of the body and reproductive organ but testis weight was maintained. It was also observed reduction of testosterone and LH levels, seminiferous tubular diameter, sperm motility and sperm counts in the epididymis. In addition, there was an increase in morphological abnormalities on spermatozoa as well as in oxidative stress level. Vitamin C reduced the oxidative stress level, diminished the number of abnormal sperm, and increased testosterone and LH levels and seminiferous tubular diameter but did not show improvement of sperm motility in relation to the hyperglycemic control group. Hyperglycemia caused a rearrangement in the epididymal tissue components (stroma, ephitelium and lumen) as demonstrated by the stereological analysis results. However, this alteration was partially prevented by vitamin C treatment.

**Conclusions:**

We conclude that vitamin C partially attenuated some male reproductive system dysfunctions in hyperglycemic rats.

## Background

Male sexual dysfunctions are frequently associated with hyperglycemia in experimental rats [1] and in men [2]. It is well known that in streptozotocin-induced hyperglycemic male rats, the reproductive changes include reduction of male organ and body weights [3,4] oligospermia [3,5,6], diminished fertility [7], decreased testosterone and gonadotropin levels [6,8] depletion of spermatogenesis and testicular damage [9,10]. Another usual consequence of hyperglycemia is the increased oxidative stress which is extremely toxic to cells and exerts its devastating effects by directly damaging cellular proteins, lipids, and DNA, or indirectly, by affecting normal cellular signaling and gene regulation [11,12].

Vitamin C, or ascorbic acid, is an important antioxidant substance in biological systems [13]. It is a water-soluble micronutrient, well absorbed by the gastrointestinal tract and required for multiple biological functions and biochemical reactions in humans and animals and it is an important element for the body [14]. In addition, it is an essential nutrient for the biosynthesis of collagen, L-carnitine and norepinephrine. As humans are unable to synthesize vitamin C, they need this vitamin in their diet. Thus, prolonged deprivation of vitamin C generates defects in the post-translational modification of collagen, followed by illness and eventually death. Low levels of vitamin C are known to occur in several pathologies which cause increased oxidative stress, such as diabetes mellitus, cancer, cataract, HIV infection, and smoking habits [13]. In the male reproductive system, vitamin C is known to protect spermatogenesis and it plays a major role in semen integrity and fertility both in men [15,16] and animals, increases testosterone levels [17] and prevents sperm agglutination. It is an important chain-breaking antioxidant, contributing up to 65 percent of the total antioxidant capacity of seminal plasma found intracellulary and extracellulary [18]. Shrilatha and Muralidhara [19] reported the protective effect of vitamin C on testicular oxidative stress, sperm oxidative stress and genotoxic effects using a diabetic mice model. Similarly, Naziroğlu [20] concluded that vitamin C acted as antioxidant in reproductive milieu. However, neither these studies nor any other evaluated the effects of vitamin C on the male reproductive system morphophysiology of hyperglycemic adult rats as shown in the current study.

Therefore, because of the clinical relevance of this matter and the lack of pertinent information in scientific literature, the present study aims to ascertain whether vitamin C could attenuate the damage in male reproductive system caused by hyperglycemia in adult male rats.

## Methods

### Animals

Adult male Wistar rats (90 days old) were supplied by Multidisciplinary Center for Biological Investigation, State University of Campinas (CEMIB - UNICAMP). During the experiment, animals were allocated into polypropylene cages (43 × 30 × 15 cm), with laboratory grade pine shavings as bedding. Rats were maintained under controlled temperature (± 23°C) and lighting conditions (12 L, 12D photoperiod, lights switched off at 07:00 am). Rat chow and filtered tap water were provided ad libitum. Experimental procedures were in accordance with the Ethical Principles in Animal Research adopted by the Brazilian College of Animal Experimentation and were approved by the Biosciences Institute/UNESP Ethics Committee for Animal Research (protocol number 022/06).

## Experimental design

### Hyperglycemic model

Severe diabetes in rats, which reproduces uncontrolled type 1 diabetes in humans, was chemically induced using a single dose of 40 mg/kg BW streptozotocin (SIGMA Chemical Company, St. Louis, MO) injected into the tail vein of adult male rats (n = 40). Streptozotocin was diluted in citrate buffer 0.01 M, pH = 4.6. Five days after the induction, glucose levels of all animals were assessed using glucose test strips and a monitoring System (One Touch Ultra, Johnson&Johnson^®^). Rats with blood glucose levels higher than 300 mg/dL were considered to be hyperglycemic, as previous described by Guneli and colleagues [10]. These animals were randomly assigned to two experimental groups of 10 animals each: hyperglycemic control (Hy) and hyperglycemic + 150 mg/day of vitamin C (HyC). This dose was adapted from Naziroğlu [20]. Another animal group (n = 10) received no streptozotocin (normoglycemic group = N group) and presented glycemic levels lower than 100 mg/dL. The vitamin C-treated hyperglycemic rats received the vitamin by gavage (oral route) throughout 30 consecutives days. Normoglycemic and hyperglycemic control groups received only the vehicle (water) during the same period. This period of treatment was chosen based on previous studies from our laboratory in which we observed that the hyperglycemic animals were not so weak until this period, which could interfere with the analysis of the reproductive system. The work was divided into two studies, denominated Experiment 1 and Experiment 2, described as follows.

### Preparation of vitamin C

Vitamin C (L-Ascorbic acid; SIGMA-ALDRICH, St. Louis, MO, USA) was prepared daily by diluting the required quantity in the corresponding volume of warm water and stored in a dark container to protect against light.

## Experiment 1

### Body weight and weight of some reproductive organs

At the end of the treatment, rats (without fasting) were slightly anesthetized with sodium pentobarbital (0.1 mL/kg weight - Hypnol^® ^3%; SYNTEC, São Paulo, Brazil), weighed and killed by decapitation. Blood was collected (between 9:00 and 11:30 am) from the ruptured cervical vessels for determination of sexual hormones and oxidative stress levels. The right testis and epididymis, ventral prostate and seminal vesicle (without the coagulating gland and full of secretion) were removed and their weights (absolute and relative to body weights) were determined. Testis and epididymis were used for sperm counts.

### Oxidative stress status assay

Blood was collected into anticoagulant tubes (Liquemine - Hoffman-La Roche, Switzerland) and was centrifuged at 90 g for 10 minutes at room temperature for assay of oxidative stress status. Hemoglobin levels were analyzed to calculate final values of oxidative stress biomarkers: SOD (superoxide dismutase), GSH-t (total glutathione), glutathione peroxidase (GSH-Px) and TBARS (thiobarbituric acid reactive species) [21]. All these biomarkers were estimated in the washed erythrocytes. Briefly 1.0 mL of washed erythrocytes were added to the test tube containing 1.0 mL of 3.0% sulphosalicylic acid, agitated for 10 seconds, centrifuged at 11.000 rpm for 3 minutes and rested for 15 minutes. The sample was diluted to 500 μl of 0.67% TBA solution (Thiobarbituric acid; SIGMA, St. Louis, USA). The mixture was heated to 80°C for 30 minutes and its absorbance measured at a wavelength 535 nm. The results were expressed as nmol of TBARS per gram of hemoglobin (nmol/g Hb), and TBARS was evaluated as an index of lipid peroxidation [21].

Superoxide dismutase (SOD) activity was determined from its ability to inhibit the auto-oxidation of pyrogallol (Pyrogallic acid; Synth^® ^, Diadema, Brazil). The reaction mixture (1.0 mL) consisted of 5.0 mmol/L Tris (hydroxymethyl) aminomethane (pH 8.0), 1.0 mmol/L EDTA, bidistilled water and 20 μL of the sample. The reaction was initiated by the addition of pyrogallol (final concentration of 0.2 mmol/L), and the absorbance was measured by a spectrophotometer with a wavelength of 420 nm (25°C) for 5 minutes. Enzymatic activity unit was defined as SOD units able to produce 50% of pyrogallol oxidation inhibition. All data were expressed in units of SOD per milligram of hemoglobin (UI/mg Hb) [21].

Glutathione total content (GSHt), which consists of reduced and oxidized glutathiones, was enzymatically determined using 5,5'-dithiobis-2-nitrobenzoic acid (DTNB; SIGMA, St. Louis, USA) and glutathione reductase in the presence of NADPH, forming 2-nitro-5-thiobenzoic acid. A mixture consisting of 1290 μL of distilled water, 200 μL of Tris/HCl buffer (1 mol/L, pH8.0; 5 mmol/L EDTA), 200 μL of 10 UI/mL glutathione reductase (GSH-r; SIGMA, St. Louis, USA), 200 μL of 2 mmol/L NADPH (SIGMA, St. Louis, USA) and 100 μL of 12 mmol/L of DTNB (SIGMA, St. Louis, USA) was added to 10 μL of the sample. Activity was measured at 412 nm on a spectrophotometer. One unit of activity was equal to micromolar of substrate reduced per gram of hemoglobin (μmol/g Hb)[21].

Glutathione peroxidase enzyme activity (GSH-Px) was measured by NADPH oxidation assay. The mixture consisted in the addition of 1300 μL of distilled water, 200 μL of Tris/HCl buffer (EDTA 1 mol/L; pH 8.0; 5 mmol/L), 200 μL of 10 UI/mL of glutathione reductase (GSH-Rd; SIGMA, St. Louis, USA), 200 μL of NADPH (2 mmol/L), 40 μL of GSH (0.1 mol/L) to 40 μL of hemolysate. The mixture was agitated in a vortex mixer for 10 seconds. Next, 20 μL of T-butyl hydroperoxide (7 mmol/L) were added and maintained at 37°C for 10 minutes. Absorbance was determined by a spectrophotometer with a wavelength of 340 nm. GSH-Px activity was expressed in enzymatic activity units per gram of hemoglobin (UI/g Hb) [21].

### Plasma testosterone, LH and FSH levels

Blood was collected into anticoagulant tubes (Liquemine - Hoffman-La Roche, Switzerland) and was used to determine plasma testosterone, luteinizing hormone (LH) and follicle-stimulating hormone (FSH) levels. The plasma was obtained by centrifugation (2400 rpm, for 20 minutes at 3.5°C) in a refrigerated centrifuge and was frozen at -20°C until the moment of hormonal determination. The analyses were determined by the technique of double antibody radioimmunoassay at the Neuroendocrinology Laboratory, Dental School of University of São Paulo - USP campus at Ribeirão Preto, Sao Paulo state, Brazil. Testosterone doses were accomplished by using the TESTOSTERONE MAIA^® ^kit (Biochem Immuno System). The LH and FSH doses used specific kits supplied by the National Institute of Arthritis, Diabetes and Kidney Diseases (NIADDK, USA). All samples were dosed in the same assay to avoid inter-assay errors. The lowest detection limits and intra-assay errors for FSH, LH and testosterone were respectively: 0.09, 0.04 and 0.064 ng/mL and 2.8, 3.4 and 4.0%.

### Daily sperm production per testis, sperm number and transit time in the epididymis

Homogenization-resistant testicular spermatids (stage 19 of spermiogenesis) in the testis were counted as described previously by Robb et al. [22], with adaptations adopted by Fernandes et al. [23]: briefly, the right testis, decapsulated and weighed soon after collection, was homogenized (T18 Ultra Turrax^® ^Homogenizer - IKA^® ^Labortechnik, Germany) in 5 mL of NaCl 0.9% containing TritonX100 0.5% (SIGMA, St. Louis, USA), followed by sonication (U50 control, ultra sonic processor - IKA^® ^Labortechnik, Germany) for 30 seconds. After a 10-fold dilution, one sample was transferred to Newbauer chambers (Neubauer improved, bright line - LABOROPTIK) and late spermatids were counted (four fields per animal). To calculate daily sperm production (DSP), the number of homogenization-resistant spermatids was divided by 6.1 (the number of days these spermatides are present in the seminiferous epithelium). In the same manner, right epididymidis portions (caput/corpus and cauda) were cut into small fragments with scissors and homogenized, and sperm counted as described for the testis. The sperm transit time through the epididymis was determined by dividing the number of sperm in each portion by DSP.

### Sperm morphology

Sperm were removed from the right vas deferens by internal rinsing with 1.0 mL of saline formol, with the aid of a syringe and needle. To analyze sperm morphology, smears were prepared on histological slides that were left to dry for 90 minutes and observed in a phase-contrast microscope (Leica DMLS) at 400× magnification [24]. One hundred spermatozoa were analyzed per animal. Morphological abnormalities were classified into two general categories pertaining to head morphology (without curvature, without characteristic curvature, or isolated form, i.e., no tail attached) and tail morphology (broken, isolated, i.e., no head attached, or rolled into a spiral [25]. This analysis was performed as described by Fernandes and colleagues [23].

## Experiment 2

Other 30 animals were randomly assigned to three groups (n = 10/group) following the same experimental protocol described for Experiment 1.

### Morphometric and histopathological analyses

The left testis and epididymis (five per experimental group) were removed and fixed in an Alfac fixing solution (80% ethanol, formaldehyde and glacial acetic acid, 8.5:1.0:0.5 v/v) for 24 h. The pieces were embedded in paraffin wax and sectioned at 7 μm. Testis and epididymis sections were stained with hematoxylin and eosin (HE) and examined by light microscopy for general histopathological and morphometric analysis as follows:

#### Seminiferous tubule diameters

Seminiferous tubule diameters were measured using Leica Q-win software (version 3 for Windows™). For this, 10 random testicular cross-sections (stage IX of the seminiferous epithelium cycle) per animal were examined blindly at 2007× magnification in a Leica microscope DMLB. In each seminiferous tubule the mean of four measures was calculated and used in the statistical analysis.

#### Spermatogenesis kinetics

Two hundred random tubular sections per animal in three nonconsecutive testis cross-sections were classified into four categories: stages I-VI, VII-VIII, IX-XIII and XIV of the seminiferous epithelium cycle, according to Leblond and Clemont [26], under a light microscope (Zeiss, Axiostar plus) at 200X magnification.

#### Epididymal stereological analysis

The stereological analysis of the epididymis was measured using Leica Q-win software (version 3 for Windows™) and a Leica microscope DMLB (200× magnification). For this, 10 random epididymal cross-sections per animal were captured and analyzed by the stereological method. This analysis was performed by means of Weibel's multipurpose graticulate, with 120 points and 60 test lines [27] to compare the relative proportion among the epididymal components (epithelium, stroma and lumen) in the experimental groups.

### Sperm motility

Sperm were obtained, from the right vas deferens, with the aid of a syringe and needle, through internal rinsing with 1.0 mL of modified HTF medium with gentamicin (Human Tubal Fluid, IrvineScientific^®^), at 34°C. A Makler counting chamber (Sefi-Medical, Haifa, Israel) warmed to 34°C was loaded with a small aliquot of sperm solution (10 μl). Sperm motility evaluation was performed by the same person throughout the study and was assessed by visual estimation (100 spermatozoa per animal, in duplicate) under a phase-contrast microscope (Leica DMLS) at 200× magnification. Spermatozoa were classified as: mobile with progressive movement, mobile without progressive movement or immobile. This analysis was performed as described by Perobelli and colleagues [28].

### Statistics

The variance among the experimental groups was compared by ANOVA, with the *post hoc *Tukey test or the non-parametric Kruskall-Wallis test, with the *post hoc *Dunn test, according to the dada distribution. Differences were considered significant when *p *< 0.05. The statistical analyses were performed by GraphPad InStat (version 3.02).

## Results

### Glycemia levels and animal health

**Figure 1:**
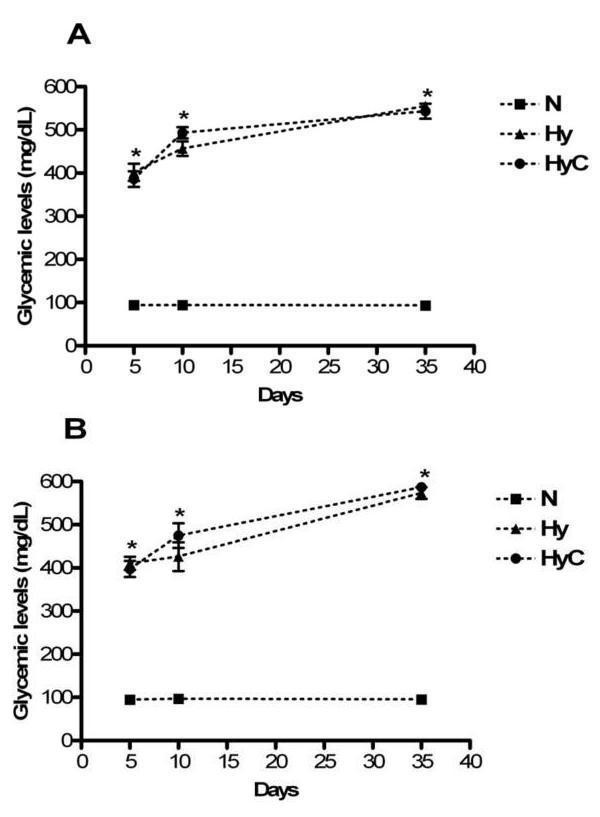
**Blood glycemic levels from Experiment 1 (A) and Experiment 2 (B)**. Values expressed as mean and S.E.M (vertical bars). Kruskal-Wallis with *post hoc *Dunn test; * p < 0.05 = Hyperglycemic groups compared with normoglycemic group. *N *= normoglycemic group; *Hy *= hyperglycemic control group; *HyC *= hyperglycemic group + vitamin C.

In both experiments, five and ten days after the hyperglycemia induction, all streptozotocin-treated animals showed glycemic levels above 300 mg/dL and were, therefore, considered hyperglycemic. These animals exhibited characteristic qualitative signs of hyperglycemia such as polyphagia, polydipsia and polyuria (data not shown). The normoglycemic rats showed normal glycemic levels, below 100 mg/dL. These glycemic statuses were maintained throughout the experimental period (Figure 1). Some hyperglycemic animals died during experimental period (Experiment 1 = one animal from vitamin C-treated hyperglycemic group; Experiment 2 = one animal from each hyperglycemic group)

### Oxidative stress analysis

In relation to oxidative stress analysis on erythrocytes (Table 1), the lipid peroxidation level (TBARS) was significantly increased in the hyperglycemic control group in relation to the other groups. The treatment with vitamin C reduced this damaging event. The hyperglycemic control group showed higher SOD activity compared to the other groups. This enzymatic activity was recovered by vitamin C in the hyperglycemic rats. Concentrations of GSHt were similar between hyperglycemic control and normoglycemic groups but these concentrations in the vitamin C-treated hyperglycemic group were significantly increased in relation to the other groups. There was a decrease of the GSH-Px activity in the hyperglycemic control group in relation to the other groups. The activity of this enzyme was maintained in the vitamin C-treated hyperglycemic rats and it was similar to the normoglycemic group.

**Table 1:** Blood biomarkers of oxidative stress

Groups	Normoglycemic (n = 10)	Hyperglycemic (n = 10)	Hyperglycemic + vitamin C (n = 9)
**TBARS (nmol/g Hb)**	39.04 [32.82 - 52.82]^a^	456.78 [391.30 - 630.00]^b^	96.60 [75.02 - 132.20]^a^
**SOD (UI/mg Hb)**	1842.80 [1434.73 - 3481.02] ^a^	3929.30 [3687.09 - 5044.50] ^b^	2000.00 [1603.20 - 2257.96]^a^
**GSHt (μmol/g Hb)**	0.05 [0.04 - 0.07] ^a^	0.08 [0.04 - 0.12] ^a^	0.29 [0.23 - 0.33] ^b^
**GSH-Px (UI/g Hb)**	0.33 [0.28 - 0.65] ^a^	0.02 [0.01 - 0.09] ^b^	0.13 [0.13 - 0.16] ^a^

Values expressed as median [Q_1 _- Q_3_]. ^a, b ^Different letters indicate groups that differ statistically (p < 0.05). Kruskal - wallis test, with the *post hoc *Dunn test.

### Body and reproductive organ weights

Body and reproductive organ weights are shown in Table 2. Streptozotocin-induced hyperglycemia produced a significant decrease in the final body, prostate and full seminal vesicle weights compared to normoglycemic animals. However, the absolute testicular and relative epididymal weights were similar among the three groups, and relative weight of ventral prostate was partially recovered in the vitamin C-treated group. There was an increase in the testis relative weight of the two hyperglycemic groups probably due to the reduced body weights of these animals.

**Table 2:** Reproductive organs and body weights

	Normoglycemic (n = 10)	Hyperglycemic (n = 10)	Hyperglycemic + vitamin C (n = 9)
**Initial BW (g)**	366.00 ± 7.75 ^a^	365.00 ± 5.80 ^a^	366.80 ± 11.26 ^a^
**Final BW (g)**	417.40 ± 9.02 ^a^	303.40 ± 9.80 ^b^	303.44 ± 7.27 ^b^
**Testis (g)** **Testis (g/100 g)**	1.80 ± 0.05 ^a^ 0.43 ± 0.01 ^a^	1.64 ± 0.05 ^a^ 0.54 ± 0.01 ^b^	1.63 ± 0.06 ^a^ 0.54 ± 0.01^b^
**Epididymis (mg)** **Epididymis (mg/100 g)**	569.84 ± 13.33 ^a^ 137.00 ± 3.70 ^a^	417.80 ± 26.37 ^b^ 137.00 ± 6.11 ^a^	436.11 ± 20.90 ^b^ 143.32 ± 4.60 ^a^
**Seminal vesicle full (g)** **Seminal vesicle full (g/100 g)**	1.37 ± 0.07 ^a^ 0.32 ± 0.02 ^a^	0.54 ± 0.12 ^b^ 0.14 ± 0.02 ^b^	0.58 ± 0.10 ^b^ 0.15 ± 0.04 ^b^
**Ventral prostate (mg)** **Ventral prostate (mg/100 g)**	418.91 ± 18.50 ^a^ 97.61 ± 4.80 ^a^	198.87 ± 19.62 ^b^ 64.82 ± 5.65 ^b^	239.38 ± 26.64 ^b^ 77.81 ± 7.31 ^ab^

Values expressed as Mean ± SEM. ^a, b ^Different letters indicate groups that differ statistically (p < 0.05). ANOVA test, with the *post hoc *Tukey test.

### Hormonal analysis

The FSH levels were unaffected by hyperglycemia. There was a significant reduction in plasma testosterone and LH levels in the hyperglycemic control group. However, in the vitamin C-treated hyperglycemic group, androgen and LH levels were partially recovered (Table 3).

**Table 3:** Hormone analysis

Groups	Normoglycemic (n = 10)	Hyperglycemic (n = 10)	Hyperglycemic + vitamin C (n = 9)
**Testosterone (ng/mL)**	1.65 [1.60 - 1.77] ^a^	0.32[0.15 - 0.85] ^b^	0.48[0.26 - 1.20] ^ab^
**LH (ng/mL)**	6.60[5.20 - 10.04] ^a^	3.03 [2.45 - 4.03] ^b^	3.60[2.75 - 4.21] ^ab^
**FSH (ng/mL)**	6.70[4.90 - 8.61] ^a^	5.30 [4.04 - 8.41] ^a^	7.10 [6.62 - 7.40] ^a^

Values expressed as median [Q_1 _- Q_3_]. ^a, b ^Different letters indicate groups that differ statistically (p < 0.05). Kruskal - Wallis test, with the *post hoc *Dunn test.

### Sperm parameters

The three groups didn't differ in relation to daily sperm production, frequency of spermatogenesis stages, absolute number of sperm and sperm transit time in the caput/corpus epididymis (data not shown). Compared to normoglycemic group, the hyperglycemic control group presented lower absolute number of sperm (millions/epididymis) and transit days in the cauda epididymis indicating an acceleration of the sperm transit time through epididimal cauda. In the vitamin C-treated group these parameters were similar to the other groups (Table 4).

**Table 4:** Sperm counts in the epididymis

Groups	Normoglycemic (n = 10)	Hyperglycemic (n = 10)	Hyperglycemic + vitamin C (n = 9)
**Sperm number in the cauda epididymis (×10^6^)**	111.00 [90.00 - 131.00] ^a^	40.16 [33.05 - 71.00] ^b^	43.00 [42.63 - 73.80] ^ab^
**Sperm transit time in the cauda (days)**	3.43 [3.30 - 4.65] ^a^	1.56 [1.25 - 2.70] ^b^	2.14 [1.72 - 2.88] ^ab^

Values expressed as median [Q_1 _- Q_3_]. ^a, b ^Different letters indicate groups that differ statistically (p < 0.05). Kruskal - Wallis test, with the *post hoc *Dunn test.

Morphology assessment of spermatozoa (Table 5) indicated that the abnormal percentages in sperm were higher in the hyperglycemic groups (Hy = 35%, HyC = 23%) compared to the normoglycemic group (9%). Vitamin C partially recovered the sperm morphology since the number of sperm with head without curvature was totally recovered and the number of sperm with isolated head and isolated tail was partially recovered by the treatment with vitamin C. The other abnormalities, as described in the methodology, were not found.

**Table 5:** Sperm morphology

Groups	Normoglycemic (n = 10)	Hyperglycemic (n = 10)	Hyperglycemic + vitamin C (n = 9)
**Head isolated**	4.70 ± 0.52 ^a^	20.00 ± 3.60 ^b^	14.00 ± 2.60 ^ab^
**Head without curvature**	1.40 ± 0.30 ^a^	3.80 ± 0.73 ^b^	1.30 ± 0.36 ^a^
**Tail isolated**	2.30 ± 0.65 ^a^	11.00 ± 3.06 ^b^	7.71 ± 1.15 ^ab^
**Normal sperm**	91.30 ± 1.03 ^a^	65.30 ± 5.05 ^b^	77.30 ± 2.24 ^b^

Values expressed as mean ± SEM. ^a, b ^Different letters indicate groups that differ statistically (p < 0.05). ANOVA test, with the *post hoc *Tukey test.

The results of sperm motility, shown in Table 6, indicated that the number of sperm with progressive movement was reduced and the number of immobile sperm was increased in the hyperglycemic groups compared to the normoglycemic group. Comparing with the normoglycemic group, the treatment with vitamin C diminished this damage since in the hyperglycemic control group the incidence of immobile sperms was more significant (p < 0.001) than in the group treated with vitamin C (p < 0.05).

**Table 6:** Sperm motility

Groups	Normoglycemic (n = 10)	Hyperglycemic (n = 9)	Hyperglycemic + vitamin C (n = 9)
**Mobile with progressive movement**	68.4 [64.25 - 69.75] ^a^	36.2 [28 - 46] ^b^	35 [17.25 - 51.75] ^b^
**Mobile without progressive movement**	8.5 [7 - 12.5] ^a^	6.0 [4 - 12] ^a^	9.0 [5.75 - 25.5] ^a^
**Immobile sperm**	22.1 [19.25 - 24.5] ^a^	56.3 [46 - 65] ^b^	47.5 [38.75 - 50.25] ^b^

Values expressed as median [Q_1 _- Q_3_]. ^a, b ^Different letters indicate groups that differ statistically (p < 0.05). Kruskal - Wallis test, with the *post hoc *Dunn test.

### Seminiferous tubular diameters, histopathology and epididymal stereological analysis

The seminiferous tubular diameters were significantly smaller in hyperglycemic control rats (Hy = 288.41 ± 3.70 - p < 0.05) in relation to the normoglycemic group (N = 303.00 ± 4.01); in the vitamin C-treated group (HyC = 295.50 ± 4.00) this parameter was considered similar to the other two groups.

However, the histopathology of the testis and epididymis (data not shown) was not affected by hyperglycemia or hyperglycemia plus vitamin C. In the testis, the spermatogenic cells, Sertolli cell and the arrangement of the seminiferous epithelium were structurally normal in the experimental groups. The Leydig cells and interstitial connective tissue seemed to be uniform in size and shape in the three groups. In the epididymis, the stromal and ephithelial tissues appeared to be normal, as well as in the lumen which showed only sperms.

The epididymal stereological analysis (Table 7) showed a significant increase of epididymal stromal compartment in animals from the hyperglycemic control group compared to normoglycemic rats, but there was a complete recovery of this compartment in the group treated with vitamin C. In both hyperglycemic groups, the epididymal duct epithelium was higher than in normoglycemic animals. This analysis also revealed a significant decrease in the epididymal luminal compartment in the hyperglycemic control group compared to normoglycemic group, but it was also totally recovered in the vitamin C-treated rats.

**Table 7:** Epididymal stereological analysis

	Experimental groups		
	Normoglycemic	Hyperglycemic	Hyperglycemic + vitamin C
**Cauda epididymis (region 6A)**			
**Stroma**	15.75 [10.71 - 22.17] ^a^	23.51 [20.39 - 26.80] ^b^	14.30 [11.01 - 20.10] ^a ^
**Epithelium**	14.28 [11.16 - 17.85] ^a^	20.24 [16.82 - 22.02] ^b^	19.64 [17.41 - 23.00] ^b^
**Lumen**	67.86 [64.29 - 75.16] ^a^	56.25 [48.51 - 62.65] ^b^	63.10 [58.63 - 70.24] ^a^

N = 50 histological fields/group. Values expressed as Median (Q_1 _- Q_3_). ^a, b ^indicate statistically different results (p < 0.05). Kruskal - Wallis test, with the *post hoc *Dunn test.

## Discussion

As previously showed [29], our study demonstrated that oral administration of vitamin C produced an antioxidant effect in the erythrocytes, as evidenced by significant increase of GSHt concentration and GSH-Px activity, and reduced TBARS levels in streptozotocin-induced hyperglycemia in rats. These results showed that oxidative stress level was reduced and also provided evidence that vitamin C may have a therapeutic role in reactive oxygen species mediated diseases.

We can suggest that the reduction of oxidative stress in the erythrocytes may also have occurred in the organs of male reproductive system and sperm, since previous studies reported that vitamin C attenuated concomitantly the oxidative stress in different tissues of hyperglycemic rats [29,30]. Thus, further studies with the same experimental design of the present study should be conducted to confirm this hypothesis.

Reductions of body, testicular, epididymal and prostate weights, shown in this study, have already been described in hyperglycemic animals [1,3,5] and can be related to the hormonal and metabolic changes that are found in hyperglycemic animals and human beings. In rats, under hyperglycemic conditions, the insulin replacement restores body and reproductive organ weights, but prostate weight can only be restored by testosterone therapy [31].

It is known that vitamin C plays key roles in the synthesis of testosterone [17,32] and in our study vitamin C-treated hyperglycemic rats showed partial recovery of testosterone level but it was not enough to prevent the loss of ventral prostate or seminal vesicle absolute weights. In this same group the ventral prostate relative weight was partially restored probably due to changes in body weight. In the present study, the reduced LH level corroborates previous studies that reported diminished LH release from pituitary gland in hyperglycemic male rats [6,33]. The partial recovery of LH levels in the vitamin C treated group may be related to the fact that ascorbic acid can be a vitaminergic transmitter that activates the release of LH and FSH from the anterior pituitary gland [34]. Although FSH levels were not reduced in the hyperglycemic rats, the normal levels of this hormone are in accordance with the sperm counts in testis and the frequencies of spermatogenesis stages. However, plasma levels of FSH were reduced in hyperglycemic rats [33] and diabetic patients [35].

Despite changes in testosterone levels in the hyperglycemic groups, the testicular weight, frequency of spermatogenesis kinetics and the daily sperm production were not affected. However, Hassan and colleagues [5] reported the reduced sperm number in the testis in diabetic rats, after six weeks of streptozotocin-induced hyperglycemia. The reduction of seminiferous tubular diameter corroborates other authors that also found this impairment in streptozotocin-induced hyperglycemia after three [36] and twenty days [10], or six weeks [1,37] and six months [9]. In contrast, Ballester and colleagues [38] reported similarity in the seminiferous tubular diameters in hyperglycemic and normoglycemic rats, after three months of streptozotocin-induced hyperglycemia. Vitamin C attenuated the reduction of seminiferous tubular diameters probably because this vitamin may have acted in the synthesis of extracellular matrix components, as previously reported [13,14], although high glucose concentration inhibits the action of vitamin C on collagen and proteoglycans synthesis [39]. It is important to realize that, there is a wide variation in the development of significant testicular atrophy among diabetic rats depending on the route of streptozotocin-administration, dose, degree of hyperglycemia, testosterone levels and most important, sampling time. Based on these arguments, the absence of testicular histopathologycal alterations may be explained, since previous studies reported histopathologycal changes in the testis [10,36,40] only after longer periods of spontaneous or streptozotocin-induced hyperglycemia. However, in short periods of hyperglycemia (15 days) such testicular alterations were not observed [3].

The decrease in absolute weight of epididymis in the hyperglycemic groups was related to a significant acceleration of the sperm transit time caused by the decrease in sperm reserves in the epididymal cauda, and by a rearrangement of the tissue components as demonstrated by the stereological analyze. Scarano and colleagues [3] reported epididymal oligospermia in streptozotocin-induced hyperglycemia and Sönmez and colleagues [17] showed that epididymal sperm concentration increased in the ascorbic acid treated rats, as evidenced in the current study. Soundamani and colleagues [41] reported that the streptozotocin-induced hyperglycemia in prepubertal rats (40 days old), caused epididymal regression in puberty (61^st ^day of postnatal life), leading to a decrease in the absolute weights of caput, corpus, and caudal regions, and a considerable reduction in tubule and lumen sizes of these segments with an increase in interstitial stroma.

In the present study, despite these stereological alterations there were no histopathological changes, corroborating previous study from our lab [3]. The regression in epididymal tissue was attenuated in the vitamin C-treated hyperglycemic group. Thus, it is quite likely that vitamin C may have specific effect on epididymal tissue. However, the mechanisms, through which vitamin C influences this tissue, are not known and there are no previous studies on this subject.

Hyperglycemic rats showed impairment in sperm morphology corroborating a study of Navarro-Casado et al., [37], and some of the abnormalities were attenuated by vitamin C treatment. This result may suggest that vitamin C could have an effect on spermiogenesis process and it could favor, at least partially, normal sperm production. The impairment in sperm motility caused by hyperglycemia also corroborated previous studies [5,7,37] but this damaged is not prevented by vitamin C. It is known that vitamin C increases gamete mobility since it reduces oxidative stress level [42]. Donnelly and colleagues [43] showed that ascorbate supplementation reduced oxidative stress, but it was not beneficial to sperm motility in human semen.

## Conclusions

In conclusion, the present study showed that vitamin C supplementation minimized some alterations in the male reproductive system caused by hyperglycemia such as reduction of testosterone and LH levels and impairment in sperm morphology. However, questions about vitamin supplementation, or any other nutrient, have been asked since excessive doses can be harmful to the body. Thus, it is possible that the beneficial effects of vitamin C supplementation are only relevant to those individuals with low levels of vitamin C and high levels of oxidative stress that occur in hyperglycemic condition. Thus, future studies are needed to complement the findings of this study.

## Competing interests

The authors declare that they have no competing interests.

## Authors' contributions

All authors participated in the design, interpretation of the studies, analysis of the data and review of the manuscript; GSAF and CDBF conducted the experiments; KEC and DCD performed biochemical analyses, JAAF performed hormone assay, and GSAF and WDGK performed data analyses and wrote the manuscript. This study represents part of GSAF Ph.D. thesis presented to the State University of Campinas, under the advisory of WDGK. All authors read and approved the final manuscript.
